# Lipid Lowering Effect of *Punica granatum* L. Peel in High Lipid Diet Fed Male Rats

**DOI:** 10.1155/2014/432650

**Published:** 2014-09-10

**Authors:** Alireza Sadeghipour, Maryam Eidi, Ali Ilchizadeh Kavgani, Reza Ghahramani, Saleh Shahabzadeh, Ali Anissian

**Affiliations:** ^1^Department of Pathology, Rasoul Akram Medical Complex, Iran University of Medical Sciences, Tehran, Iran; ^2^Department of Biology, College of Biological Sciences, Varamin-Pishva Branch, Islamic Azad University, Varamin, Iran; ^3^Young Researchers and Elite Club, Islamic Azad University, Tehran Medical Sciences Branch, Tehran, Iran; ^4^Young Researchers and Elite Club, Islamic Azad University, Varamin-Pishva Branch, Varamin, Iran; ^5^Department of Veterinary, College of Agriculture, Abhar Branch, Islamic Azad University, Abhar, Iran

## Abstract

Many herbal medicines have been recommended for the treatment of dyslipidemia. The antilipidemic effect of hydroethanolic extract of pomegranate peel (*Punica granatum* L.) was investigated in high lipid diet fed male rats. Intraperitoneally administration of pomegranate peel extract (50, 100, 200, and 300 mg/kg body weight) for 23 days on the levels of serum cholesterol, triglycerides, LDL, HDL, alkaline phosphatase (AP), aspartate aminotransferase (AST), and alanine aminotransferase (ALT) in high lipid diet fed male rats was evaluated. Treatment of pomegranate extract decreased body weight in treated rats, significantly. Administration of the plant extract significantly decreased serum total cholesterol, triglycerides, LDL-C, alkaline phosphatise, AST, and ALT levels, whereas it increased serum HDL-C in high lipid diet fed rats in comparison to saline control group. Also, histopathological study showed that treatment of pomegranate peel extract attenuates liver damage in high lipid diet fed rats in comparison to saline group. It is concluded that the plant should be considered as an excellent candidate for future studies on dyslipidemia.

## 1. Introduction

Dyslipidemia is generally characterized by elevated levels of total cholesterol, triglycerides, low density lipoprotein cholesterol, and decreased levels of high density lipoprotein cholesterol [1]. Dyslipidemia as an independent preventable risk factor of coronary heart disease has been shown to increase the risk of cardiovascular mortality [2–7]. Therefore, the study on the various indicators and risk factors of dyslipidemia appears to be significant in future health outcomes.

*Punica granatum* Linn. (Punicaceae) is a shrub or small tree and considered to be a native of Iran and Afghanistan. It is also found growing wild in the warm valleys and outer hills of the Himalayas [8]. The pomegranate fruit consists of the peel, seeds, and the arils. The peel makes up about 50% of the fruit, whereas the arils and seeds make up 40% and 10%, respectively. The peel is rich in many compounds such as phenolics, flavonoids, ellagitannins and proanthocyanidin compounds, complex polysaccharides, and many minerals including potassium, nitrogen, calcium, magnesium, phosphorus, and sodium [9].

The different parts of pomegranate (*Punica granatum* L.) have been known as a reservoir of bioactive compounds with potential biological activities. Pomegranate decreased the dyslipidemia of obesity and cardiovascular risk factors [10]. Antiparasitic, antimicrobial, and antioxidant activities of pomegranate leaves extracts were reported [11–13]. Several papers were reported on the ability of pomegranate leaves extracts to fight obesity [14], cancer, and other human diseases [15].

It is reported that 6-week treatment with pomegranate flower extract ameliorated fatty liver, reflected by diminishment of relative and total hepatic triglyceride contents and fatty droplet deposit in the livers of Zucker diabetic fatty rats [16].

In traditional Chinese medicine, different pomegranate extracts and preparations including the bark, root, and juice of the fruit, especially the dried peels, have been used to treat many conditions [10].

The aim of the present study was to investigate the antihyperlipidemic effects of pomegranate extract peel in high lipid diet fed male rats.

## 2. Materials and Methods

### 2.1. Plant Material

Fresh* Punica granatum* L. peels were collected from Saveh area (October 2013). Voucher specimens (Farabi Herbarium number GUE 7321) were authenticated by Associate Professor Ali Mazooji, Department of Biology, Faculty of Biology, Islamic Azad University. The plant material was dried under shade and powdered using Ultra-Torax. The powder (60 g) was extracted with 300 mL aqueous 80% ethanol in a Soxhlet apparatus for 72 hours. The extract was filtered and concentrated to dryness under reduced pressure in a rotary evaporator at 40–50°C yielding 15.3% (w/w) plant extract. The extract yield was 19%. The obtained pomegranate alcoholic extract was stored at −20°C until usage. Plant extract was suspended in saline (doses 50, 100, 200, and 300 mg/kg body weight) prior to intraperitoneal administration to the experimental animals.

### 2.2. Experimental Animals and Induction of Hyperlipidemia

Male Wistar rats initially weighing 200 to 250 g purchased from the Pasteur Institute (Karaj, Iran) were used in the experiments. The diet was purchased from Pars-Dam food service, Tehran, Iran. The animal room was maintained at 22°C ± 2°C with timed lighting on from 7 AM to 19 PM and relative air humidity of 40% to 60%. Each animal was used once only. The animal protocol was approved by the Ethics Committee of Islamic Azad University, Tehran, Iran, and conforms to the guidelines of the Committee for the Purpose of Control and Supervision on Experiments on Animals, Iran, and also to international guidelines. Accordingly, five rats were housed per cage of size 50 cm × 23 cm. Hyperlipidemia was induced by feeding 10% lipid supplemented in the basal diet. The basal diet contained (g% of final diet), casein 15.0, sucrose 68.3, hydrogenated coconut oil 10.0, cellulose 2.0, salt mixture 4.0, vitamin mixture 0.5, and choline chloride 0.2. The animals were distributed into six groups each containing 8 rats. The control group was fed on the basal diet and given water* ad libitum*. Extract was dissolved in saline and administered intraperitoneally (i.p.) for 23 days. Animals in the control group received only 0.5 mL saline as vehicle.

Experimental groups were as follows: Group 1: normal control, fed on basal diet; Group 2: untreated control, fed on 10% lipid diet and given saline 0.5 mL/rat (i.p.); Groups 3, 4, 5, and 6, fed on 10% lipid in diet and administered extract at doses 50, 100, 200, and 300 mg/kg/day (i.p.).

The initial body weights of all the animals in each group were measured. After 23 days, the rats were fasted for 12 h and their final body weights were determined. Then, rats were fasted overnight, and blood samples were drawn from heart under light ether anaesthesia. The animals were removed after blood collection. Serum cholesterol, triglyceride, LDL, HDL, aspartate aminotransferase (AST), and alanine aminotransferase (ALT) levels were determined by kit (Parsazmoon Company, Iran).

### 2.3. Histopathological Studies in the Liver

For qualitative analysis of liver histology, the tissue samples were fixed for 48 h in 10% formalin-saline and dehydrated by passing successfully in different mixtures of ethyl alcohol-water, cleaned in xylene, and embedded in paraffin. Sections of the tissue were prepared by using a rotary microtome and stained with haematoxylin and eosin dye, which was mounted in a neutral deparaffinated xylene medium for microscopic observations. Histological damage including fatty change in hepatocyte, dilation of sinozoid, and congestion in high lipid diet fed. Each damage is given 1 score.

### 2.4. Statistical Analysis

Statistical analyses and representations were performed in Microsoft Excel. All data was analyzed by one-way ANOVA and presented as the mean value ± S.E.M. of eight rats (*n* = 8). The results of the lipid fed untreated control group were compared to normal control group and those of extract treated groups were compared to untreated control group. *P* values were checked at three levels of significance, namely, 0.05, 0.01, and 0.001. *P* value less than 0.05 was considered “significant” and *P* less than 0.01 and 0.001 as “highly significant.”

## 3. Results

### 3.1. General Improvement in the Hyperlipidemic State

**Figure 1:**
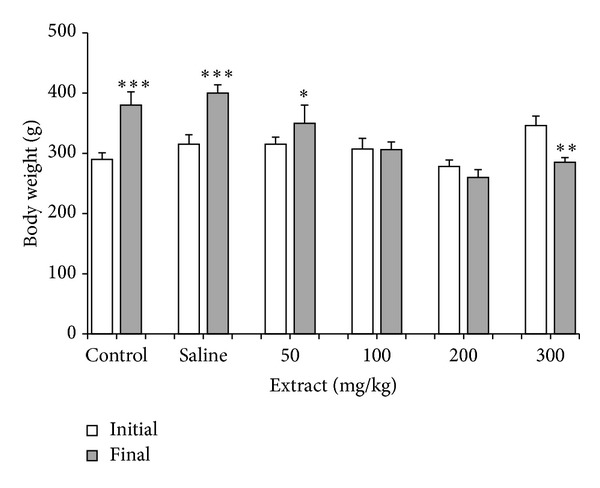
Effect of i.p. administration of pomegranate peel hydroethanolic extract at doses of 50, 100, 200, and 300 mg/kg body wt on body weight in high lipid diet fed rats. Each column represents mean ± SEM for 8 rats. Control saline group was administrated with saline as vehicle. *P < 0.05, **P < 0.01, ***P < 0.001 different from initial body weight in each group.

Changes in initial and final body weights in control and experimental groups are shown in Figure 1. The results showed treatment of extract decreased final body weight elevations in comparison to control saline group (*P* < 0.01).

The results showed treatment of extract decreased liver and kidney coefficients (liver weight/body weight and kidney weight/body weight) in comparison to control saline group, insignificantly. The administration of the pomegranate peel extract (50, 100, 200, and 300 mg/kg body wt) significantly decreased serum triglycerides, cholesterol, LDL, AP, ALT, and AST levels, while increasing serum HDL level in high lipid diet fed rats compared with saline group (Table 1).

**Figure 2:**
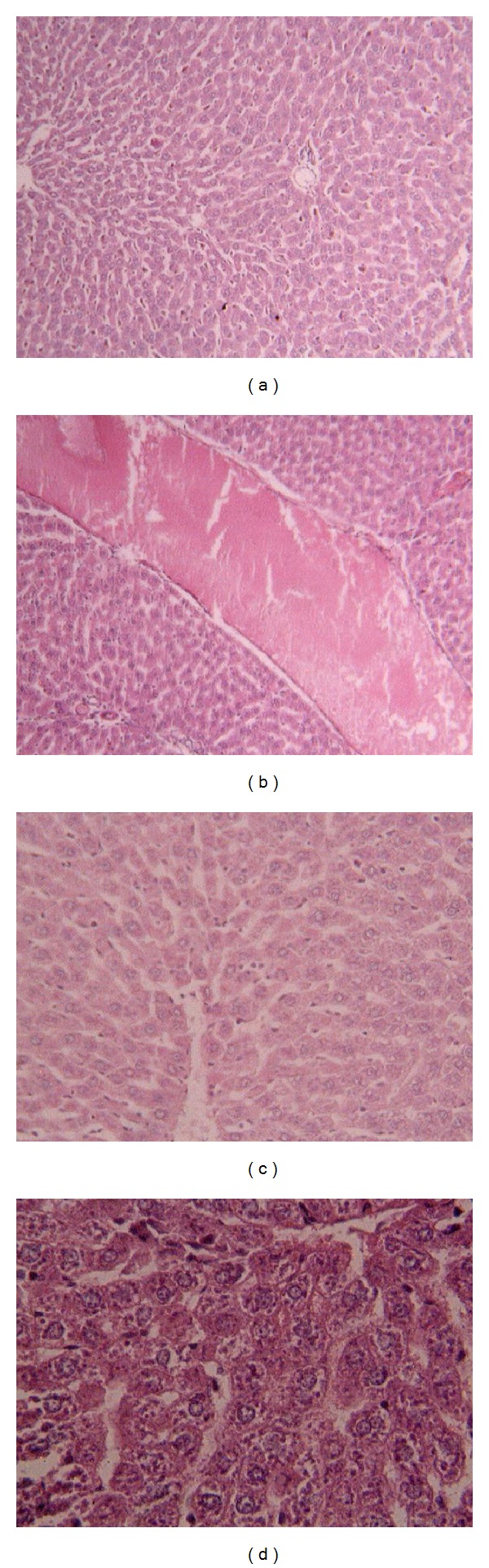
Histopathology of liver tissue in high lipid diet and normal diet fed rats (hematoxylin-eosin). (a) Control liver tissue (×100), (b) congestion damage in high lipid diet fed rats (×100), (c) dilation of sinusoid (×400), and (d) fatty change in hepatocyte (×100).

Histopathological study shows that the administration of the pomegranate peel extract (50, 100, 200, and 300 mg/kg body wt) significantly decreased histopathological damage of liver including fatty change in hepatocyte, dilation of sinusoid, and congestion (Figure 2) in high lipid diet fed rats compared with saline group (Table 1).

## 4. Discussion

Dyslipidemia is a multifactorial and polygenic disorder resulting from an interaction between an individual's genetic background and multiple environmental factors including behavioural and social risk factors [10].

Fruits are rich sources of vitamins, minerals, and biologically active compounds. However, very often they are consumed without the peels despite the fact that some fruit peels are rich in polyphenolic compounds, flavonoids, ascorbic acid, and other biologically active components that have positive influence on health [2, 17].

Our results demonstrated that administration of hydroethanolic extract from* Punica granatum* peel showed marked antihyperlipidemic effects in high lipid diet fed rats. Pomegranate extract decreased serum cholesterol, triglycerides, LDL, ALT, AST, and AP, while increasing serum HDL levels in high lipid diet fed rats in comparison to saline treated rats. Also, the extract attenuated liver damage including fatty change in hepatocyte, dilation of sinusoid, and congestion in high lipid diet fed rats compared with saline group.

In agreement, it is reported that the different parts of pomegranate (*Punica granatum* L.) have been known as a reservoir of bioactive compounds with potential biological activities. Pomegranate, especially the leaves of pomegranate, decreased the dyslipidemia of obesity and cardiovascular risk factors [10]. The ability of pomegranate leaves extracts to fight obesity is shown [14].

On the other hand, pomegranate flower has been demonstrated to ameliorate hyperlipidemia and decrease excess cardiac lipid accumulation in Zuker diabetic fatty rats [18] and to attenuate atherosclerosis in apolipoprotein E deficient mice [19]. Moreover, oleanolic acid and ursolic acid, two of the active components contained in pomegranate flower [20], have been long-recognized to have antihyperlipidemic properties [21]. Gallic acid, another important component in pomegranate flower [20], has been demonstrated to improve high fat diet induced hyperlipidemia and fatty liver in mice [22].

Also, Parmar and Kar reported pomegranate peel extract ameliorated biochemical and histopathologic alterations induced by the atherogenic diet [23]. The protective role of the fruit peel could be related to its flavonoids and polyphenolic contents, which possess antioxidative activity [24]. Moreover, the juice of* P. granatum* is also known to prevent atherosclerosis, which further supports its antiatherogenic potential [25].

It is reported that addition of pomegranate juice to simvastatin in a macrophage cell culture model system improves the statin ability to inhibit cellular cholesterol biosynthesis and to protect the cells from oxidative stress. These effects could be related to the antioxidant hydrolyzable tannin punicalagin and to the phytosterol *β*-sitosterol, which are both present in pomegranate [26]. Moreover, phytosterols of pomegranate consumption decreased serum cholesterol levels in dyslipidemic patients, as well as their cardiovascular risk [27, 28].

As a result, it may be concluded that pomegranate peel seeds extract possesses antilipidemic activities in high lipid diet fed rats and that the pomegranate peel extract may be of use as an antidyslipidemic agent. It is concluded that the plant should be considered as an excellent candidate for future studies on dyslipidemia. In addition, further comprehensive pharmacologic investigations, including experimental chronic studies, should be carried out.

## Figures and Tables

**Table 1:** Effect of i.p. administration of hydroethanolic extract of *Punica granatum* peel at doses 50, 100, 200, and 300 mg/kg on liver and kidney coefficients, serum parameters, and histopathological damage of liver in high lipid diet fed rats.

Parameters	Control	Saline	Extract (mg/kg)			
			50	100	200	300
Liver coefficient	0.028	0.042	0.037	0.035	0.039	0.039
Kidney coefficient	0.0027	0.0036	0.0033	0.0033^+^	0.0035	0.0036
Triglycerides (mg/dL)	146 ± 21	475 ± 11∗∗∗	381 ± 23	325 ± 43	302 ± 31	210 ± 27^+++^
Cholesterol (mg/dL)	73 ± 8	110 ± 11∗∗∗	87 ± 9^++^	82 ± 5^++^	80 ± 9^++^	81 ± 7^++^
LDL (mg/dL)	92 ± 6	321 ± 11∗∗	209 ± 23	145 ± 29^+^	79 ± 8^++^	61 ± 7^+++^
HDL (mg/dL)	98 ± 9	28 ± 3∗	89 ± 11	128 ± 5^+++^	179 ± 18^+++^	185 ± 20^+++^
AST (UI/L)	1234 ± 34	1538 ± 47∗∗∗	1219 ± 39^+++^	1232 ± 71^+++^	1170 ± 49^+++^	1233 ± 36^+++^
ALT (UI/L)	1267 ± 56	1553 ± 32∗∗∗	1130 ± 44^+++^	1246 ± 51^+++^	1233 ± 68^+++^	1290 ± 54^+++^
AP (UI/L)	983 ± 21	1362 ± 71∗∗∗	1311 ± 39	1248 ± 48	1100 ± 59^++^	1049 ± 78^+++^
Histopathological damage of liver	0 ± 0	1.5 ± 0.11∗	0.5 ± 0.23	0.4 ± 0.23	0.17 ± 0.31^+^	0.09 ± 0.27^+^

*P < 0.05, **P < 0.01, ***P < 0.001 different from control group.

^+^ *P* < 0.05, ^++^ *P* < 0.01, ^+++^ *P* < 0.001 different from saline group.

## References

[1] Yan-Ling Z, Dong-Qing Z, Chang-Quan H, Bi-Rong D (2012). Cigarette smoking and its association with serum lipid/lipoprotein among Chinese nonagenarians/centenarians. *Lipids in Health and Disease*.

[2] Castelli WP, Garrison RJ, Wilson PWF (1986). Incidence of coronary heart disease and lipoprotein cholesterol levels. The Framingham Study. *The Journal of the American Medical Association*.

[3] Lipid Research Clinics Program (1984). The lipid research clinics coronary primary prevention trial results. I. Reduction in incidence of coronary heart disease. *Journal of the American Medical Association*.

[4] Lipid Research Clinics Program (1984). The Lipid Research Clinics Coronary Primary Prevention Trial results. II. The relationship of reduction in incidence of coronary heart disease to cholesterol lowering. *Journal of the American Medical Association*.

[5] Okamura T (2010). Dyslipidemia and cardiovascular disease: a series of epidemiologic studies in Japanese populations. *Journal of Epidemiology*.

[6] Sharrett AR, Ballantyne CM, Coady SA (2001). Coronary heart disease prediction from lipoprotein cholesterol levels, triglycerides, lipoprotein(a), apolipoproteins A-I and B, and HDL density subfractions: the Atherosclerosis Risk in Communities (ARIC) Study. *Circulation*.

[7] Toth PP (2008). Subclinical atherosclerosis: what it is, what it means and what we can do about it. *International Journal of Clinical Practice*.

[8] Satyavati GV, Gupta AK, Tandon N (1978). *Medicinal Plants of India*.

[9] Viuda-Martos M, Fernández-López J, Pérez-Álvarez JA (2010). Pomegranate and its many functional components as related to human health: a review. *Comprehensive Reviews in Food Science and Food Safety*.

[10] Lei F, Zhang XN, Wang W (2007). Evidence of anti-obesity effects of the pomegranate leaf extract in high-fat diet induced obese mice. *International Journal of Obesity*.

[11] Egharevba HO, Kunle OF, Iliya I (2010). Phytochemical analysis and antimicrobial activity of *Punica granatum* L. (fruit bark and leaves). *New York Science Journal*.

[12] El-Shennawy A, Ali E, El-Komy W (2010). Evaluation of ponytail antiparasitic activity of pomegranate juice, peels and leaves against Giardia lamblia. *International Journal of Infectious Diseases*.

[13] Wang C, Shi L, Fan L (2013). Optimization of extraction and enrichment of phenolics from pomegranate (*Punica granatum* L.) leaves. *Industrial Crops and Products*.

[14] Al-Muammar MN, Khan FF (2012). Obesity: the preventive role of the pomegranate (*Punica granatum*). *Nutrition*.

[15] Lansky EP, Newman RA (2007). *Punica granatum* (pomegranate) and its potential for prevention and treatment of inflammation and cancer. *Journal of Ethnopharmacology*.

[16] Xu KZ, Zhu C, Kim MS, Yamahara J, Li Y (2009). Pomegranate flower ameliorates fatty liver in an animal model of type 2 diabetes and obesity. *Journal of Ethnopharmacology*.

[17] Leontowicz M, Gorinstein S, Leontowicz H (2003). Apple and pear peel and pulp and their influence on plasma lipids and antioxidant potentials in rats fed cholesterol-containing diets. *Journal of Agricultural and Food Chemistry*.

[18] Huang THW, Yang Q, Harada M (2005). Pomegranate flower extract diminishes cardiac fibrosis in zucker diabetic fatty rats: modulation of cardiac endothelin-1 and nuclear factor-kappaB pathways. *Journal of Cardiovascular Pharmacology*.

[19] Aviram M, Volkova N, Coleman R (2008). Pomegranate phenolics from the peels, arils, and flowers are antiatherogenic: studies in vivo in atherosclerotic apolipoprotein E-deficient (E0) mice and in vitro in cultured macrophages and lipoproteins. *Journal of Agricultural and Food Chemistry*.

[20] Li Y, Qi Y, Huang THW, Yamahara J, Roufogalis BD (2008). Pomegranate flower: a unique traditional antidiabetic medicine with dual PPAR-*α*/-*γ* activator properties. *Diabetes, Obesity and Metabolism*.

[21] Liu J (2005). Oleanolic acid and ursolic acid: research perspectives. *Journal of Ethnopharmacology*.

[22] Jang A, Srinivasan P, Lee NY (2008). Comparison of hypolipidemic activity of synthetic gallic acid-linoleic acid ester with mixture of gallic acid and linoleic acid, gallic acid, and linoleic acid on high-fat diet induced obesity in C57BL/6 Cr Slc mice. *Chemico-Biological Interactions*.

[23] Parmar HS, Kar A (2007). Protective role of *Citrus sinensis*, *Musa paradisiaca*, and *Punica granatum* peels against diet-induced atherosclerosis and thyroid dysfunctions in rats. *Nutrition Research*.

[24] Chidambara Murthy KN, Jayaprakasha GK, Singh RP (2002). Studies on antioxidant activity of pomegranate (*Punica granatum*) peel extract using in vivo models. *Journal of Agricultural and Food Chemistry*.

[25] Sestili P, Martinelli C, Ricci D (2007). Cytoprotective effect of preparations from various parts of Punica granatum L. fruits in oxidatively injured mammalian cells in comparison with their antioxidant capacity in cell free systems. *Pharmacological Research*.

[26] Rosenblat M, Volkova N, Aviram M (2013). Pomegranate phytosterol (*β*-sitosterol) and polyphenolic antioxidant (punicalagin) addition to statin, significantly protected against macrophage foam cells formation. *Atherosclerosis*.

[27] Rocha M, Bañuls C, Bellod L, Jover A, Víctor VM, Hernández-Mijares A (2011). A review on the role of phytosterols: new insights into cardiovascular risk. *Current Pharmaceutical Design*.

[28] Moruisi KG, Oosthuizen W, Opperman AM (2006). Phytosterols/stanols lower cholesterol concentrations in familial hypercholesterolemia subjects: a systematic review with meta-analysis. *Journal of the American College of Nutrition*.

